# The serum CXCL13 level is associated with the Glasgow Prognostic Score in extranodal NK/T-cell lymphoma patients

**DOI:** 10.1186/s13045-015-0142-4

**Published:** 2015-05-14

**Authors:** Seok Jin Kim, Kyung Ju Ryu, Mineui Hong, Young Hyeh Ko, Won Seog Kim

**Affiliations:** Division of Hematology and Oncology, Department of Medicine, Samsung Medical Center, Sungkyunkwan University School of Medicine, Seoul, Korea; Samsung Biomedical Research Institute, Samsung Medical Center, Seoul, Korea; Department of Pathology, Kangnam Sacred Heart Hospital, Hallym University, Seoul, Korea; Department of Pathology, Samsung Medical Center, Sungkyunkwan University School of Medicine, Seoul, Korea; Department of Health Sciences and Technology, SAIHST, Sungkyunkwan University School of Medicine, Seoul, Korea

**Keywords:** Extranodal NK/T-cell lymphoma, Inflammation, Chemokine, CXCL13, GPS

## Abstract

**Background:**

The Glasgow Prognostic Score (GPS) measures inflammation and proves its prognostic value in patients with extranodal natural killer (NK)/T-cell lymphoma (ENKTL) which is commonly combined with inflammatory lesion. Given inflammatory chemokines play an important role in tumor progression, we hypothesized that chemokines might influence ENKTL aggressiveness through interaction with their receptors in the tumor tissue.

**Methods:**

We measured the serum levels of C-X-C motif ligand 13 (CXCL13) in 69 patients with ENKTL who received non-anthracycline-based chemotherapy and/or concurrent chemoradiotherapy because CXCL13 is thought to have a pro-tumor effect through interaction with its receptor, the C-X-C chemokine receptor 5 (CXCR5). We analyzed the association of serum CXCL13 with the GPS, and their prognostic relevance. The levels of CXCL13 were measured using a multiplex chemokine assay on archived frozen serum samples.

**Results:**

Patients were categorized into high and low CXCL13 groups if they had CXCL13 levels above or below the median value of 29.1 pg/mL, respectively. The high CXCL13 group and grouping by the GPS showed a significant association with poor progression-free survival. The elevated serum levels of CXCL13 were also significantly associated with a high score of the GPS. High CXCL13 levels and GPS were significantly associated with high tumor burden predicting poor prognosis including stages III/IV, extranasal presentation, bone marrow invasion, and presence of Epstein-Barr virus (EBV) DNA in blood. Furthermore, serum CXCL13 and GPS discriminated patients at risk of treatment failure among patients with low tumor burden (stage I/II) and non-detectable EBV DNA.

**Conclusions:**

Serum levels of CXCL13 were associated with the prognostic value of GPS. Grouping by the serum CXCL13 might predict survival outcomes in patients with ENKTL, suggesting that it is a potential therapeutic target.

## Background

Extranodal natural killer (NK)/T-cell lymphoma (ENKTL) is a subtype of non-Hodgkin lymphoma (NHL), and ENKTL is strongly associated with latent Epstein-Barr virus (EBV) infection and usually shows aggressive behavior with a poor prognosis [1]. Although chemoradiotherapy and non-anthracycline-based chemotherapy have been shown to improve outcome [2,3], treatment failures occur in patients with any stage of disease. Identifying a prognostic indicator might allow the identification of patients who are at risk of poor treatment outcomes. A recent study demonstrated that the Glasgow Prognostic Score (GPS) has prognostic value in ENKTL [4]. The GPS score is based on measures of C-reactive protein (CRP) and albumin and therefore reflects inflammatory activity [5]. Given that GPS reflects both inflammation and ENKTL prognosis, tumor aggressiveness may be associated with higher states of inflammation. Inflammatory cells frequently infiltrate ENKTL tumors, and the majority of patients have clinical signs of inflammation such as nasal tract inflammation. However, any underlying mechanism by which inflammation may contribute to poor ENKTL prognosis is still unknown.

The inflammatory milieu of a tumor’s microenvironment is important in its growth and progression [6,7]. Therefore, we hypothesized that chemokines might influence ENKTL aggressiveness through interaction with their receptors in the tumor tissue. Chemokines are a family of small signaling cytokines that recruit leukocytes to inflammatory sites and play a role in homeostatic activities (including subsequent lymphocyte migration) [8,9]. On the other hand, chemokines also contribute to tumor development, growth, and metastasis [10,11]. Among various chemokines, C-X-C motif ligand 13 (CXCL13, also named as B-cell attracting chemokine 1 (BCA-1)) is thought to play an important pro-tumor role in colon, prostate, and breast cancers through the interaction with its receptor, the C-X-C chemokine receptor 5 (CXCR5) [12-14]. In particular, a recent *in vitro* study with colorectal cancer cell lines reported the interaction of CXCL13 with CXCR5 activated the phosphatidylinositol-3 kinase (PI3K)/AKT pathway leading to migration and invasion of cancer cells [15]. In NHL, a recent prospective study of inflammatory markers demonstrated a significant association of serum CXCL13 with the risk of lymphoma supporting its role in the lymphoma development [16]. Thus, considering its association with lymphoma and the activation of PI3K/AKT pathway, CXCL13 might have a positive correlation with the aggressiveness of ENKTL because latent membrane protein 1, an EBV oncoprotein could activate the PI3K/AKT pathway in ENKTL [17]. If so, the elevated serum level of CXCL13 could be an underlying mechanism for the prognostic value of GPS in patients with ENKTL. Therefore, we measured the serum levels of CXCL13 and analyzed their correlation with the GPS and survival outcomes of ENKTL patients.

## Patients and methods

### Patients

Patients diagnosed with ENKTL from two prospective cohort studies between September 2008 and December 2012 (first study: 2008–2011, NCT#00822731; second ongoing study since 2012, NCT#01877109) were included in this study. Written informed consent was obtained from all patients. Clinical information, laboratory results, and serum samples at diagnosis were obtained from the cohort studies. All patients had received treatment with curative intent. Treatments included concurrent chemoradiotherapy (CCRT) followed by systemic chemotherapy for stage IE or IIE as previously reported [2,18] or systemic chemotherapy with SMILE (steroid, methotrexate, ifosfamide, L-asparaginase, and etoposide) for stage III/IV [19,20]. The primary tumor site was determined based on the clinical presentation and included nasal and extranasal sites, such as the skin, soft tissue, and other organs. Three comprehensive prognostic models including International Prognostic Index (IPI), NK Prognostic Index (NKPI), and GPS were used to determine risk group stratification. The GPS was calculated according to serum CRP and albumin levels that were measured as part of the clinical practice at diagnosis as described previously [5,21]. Thus, patients with both an elevated CRP level (>10 mg/L) and hypoalbuminemia (<35 g/L) received a score of 2 whereas patients with elevated CRP or hypoalbuminemia were allocated a score of 1. Patients with neither of these abnormalities were given a score of 0. The EBV DNA titer was measured upon diagnosis from a whole blood sample, as previously reported [22]. The patients were dichotomized according to positive and negative EBV DNA. Survival status was updated at the time of analysis in December 2014. This study was approved by the Institutional Review Board of the Samsung Medical Center.

### Measurement of serum CXCL13 and CXCL13/CXCR5 immunohistochemistry

Serum samples were collected at diagnosis and stored at −80°C until analysis. A Procarta cytokine profiling kit (Panomics, CA, USA) was used to measure CXCL13 levels (three times) according to the manufacturer’s instructions. In order to evaluate the tissue expression of CXCL13 and CXCR5, immunohistochemical analysis was performed on formalin-fixed, paraffin-embedded, 4-μm thick tissue sections. The tissue sections were deparaffinized three times in xylene for a total of 15 min and were then incubated with the primary monoclonal antibody against CXCL13 (Clone 53610; MAB801; R&D Systems, Minneapolis, MN, USA), and CXCR5 (Clone 51505; FAB190P; R&D Systems, Minneapolis, MN, USA). Immunostaining was performed using a BOND-MAX autoimmunostainer (Leica Microsystems, Wetzlar, Germany) with BOND Polymer Refine Detection (DS9800; Vision BioSystems, Melbourne, Australia). Positivity for CXCL13 and CXCR5 was determined by comparing to the normal positive control from the tonsillar germinal center. In every staining set, a negative control was included in which the primary antibodies and probes were omitted.

### Statistics

The chi-square test was used to analyze the association between serum CXCL13 with clinical and laboratory parameters. The Kaplan-Meier method was used for univariate analysis of survival outcomes. The survival outcomes were compared with the log-rank test. Progression-free survival (PFS) was defined as the time from the date of diagnosis to the date of documented disease progression or death. In contrast, the overall survival (OS) was measured from the date of diagnosis to the date of death due to any cause. The OS was censored at the date of the last follow-up visit. Cox regression hazard analysis was used in multivariate analysis for survival outcomes. A two-sided *P* value <0.05 was considered statistically significant.

## Results

### Characteristics of patients

**Figure 1:**
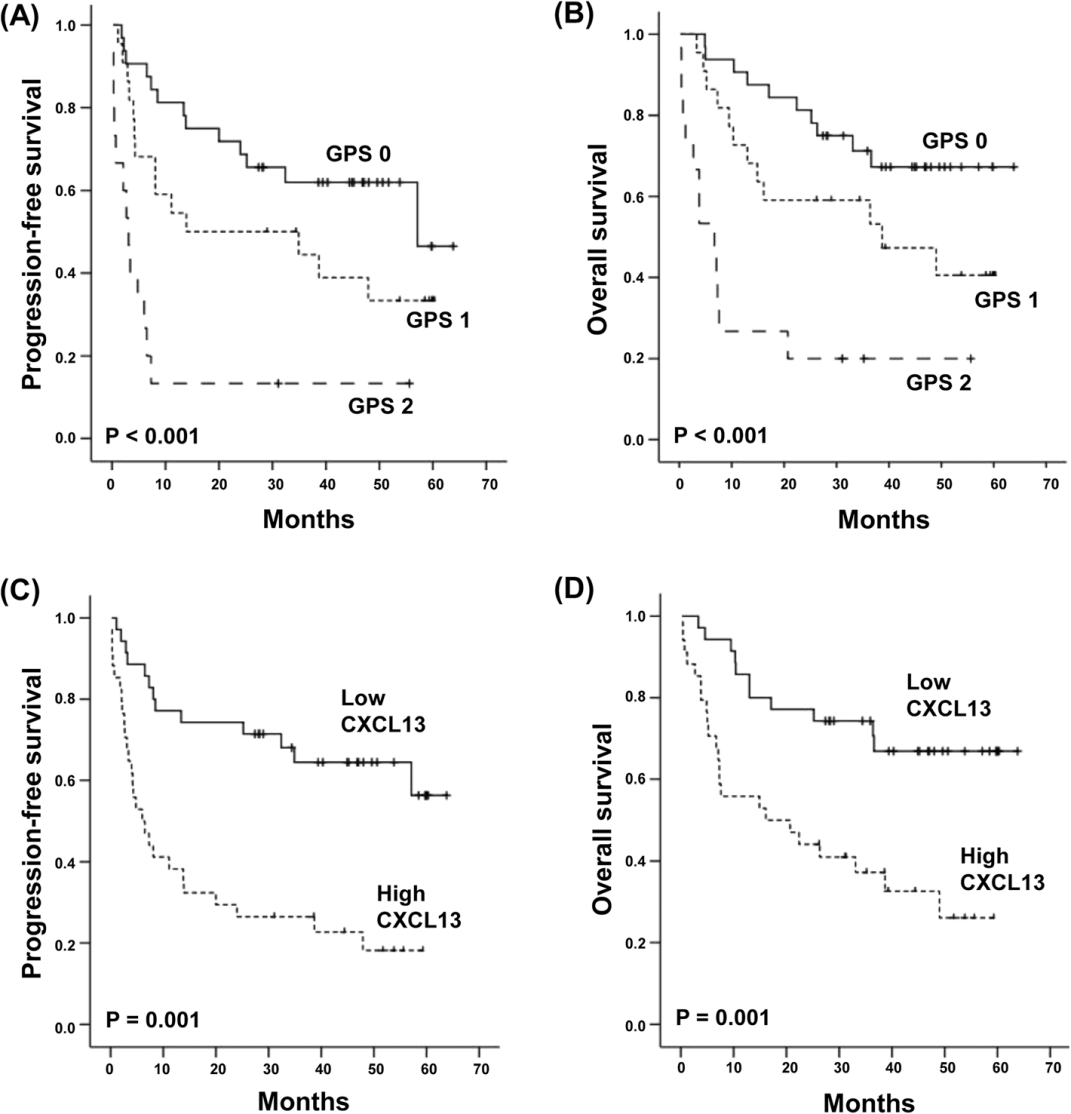
The association of overall and progression-free survival with GPS and serum CXCL13. **(A, B)** Grouping by the GPS demonstrates a significant association with OS and PFS. **(C, D)** High CXCL13 levels are more significantly associated with shorter overall and progression-free survival than are lower levels.

We analyzed 69 patients with a median age of 48 years at diagnosis (range 17–75 years). Forty patients (58%) had stages I or II, while 29 patients had stages III or IV (42%). The most common primary tumor site was the nasal tract (*n* = 41). Twenty-eight patients had extranasal disease in areas including the skin and gastrointestinal tract (Table 1). A circulating EBV DNA level was detected in the whole blood of 27 patients, while 42 patients had no detectable EBV DNA. The majority of patients (*n* = 44, 64%) were classified as being at low or low-intermediate risk based on the IPI. Forty-three patients (62%) were classified into groups III or IV based on their NKPI [23,24]. The median potential follow-up was 47 months. A total of 38 patients had relapse or progression. Thirty-four patients died, including two cases of non-disease-related death. Univariate analysis of the survival outcomes demonstrated that all clinical parameters (except sex) were significantly associated with OS and PFS (Table 1). The GPS also demonstrated prognostic relevance to OS and PFS in ENKTL patients, as previously reported (Figure 1A, B) [4].

**Table 1:** **Clinical patient characteristics and their association with survival outcomes**

				**PFS**		**OS**	
**Characteristics**		***n***	**%**	**HR**	***P***	**HR**	***P***
Age (years)	≤60	56	81				
	>60	13	19	2.164	0.039	2.489	0.024
Sex	Male	46	33				
	Female	23	67	1.215	0.575	0.752	0.494
Performance status	ECOG 0/1	56	81				
	ECOG ≥2	13	19	4.372	<0.001	3.736	0.001
Ann Arbor stage	I/II	29/11	42/16				
	III/IV	5/24	7/35	1.935	<0.001	1.898	<0.001
Serum LD	Normal	30	44				
	Increased	39	56	3.124	0.002	2.456	0.026
B symptoms	Absence	39	56				
	Presence	30	44	2.634	0.004	2.310	0.027
LN involvement	Absence	40	58				
	Presence	29	42	2.236	0.017	3.258	0.002
Primary site	Nasal	41	59				
	Extranasal	28	41	4.092	<0.001	4.240	<0.001
Bone marrow invasion	No	56	81				
	Yes	13	19	6.591	<0.001	5.298	<0.001
Extranodal involvement	0/1	43	62				
	≥2	26	38	2.948	0.001	2.616	0.011
EBV DNA	Non-detectable	42	61				
	Detectable	27	39	4.111	<0.001	3.482	<0.001
IPI	Low/low-intermediate	33/11	48/16				
	High-intermediate/High	17/8	24/12	2.482	<0.001	2.031	<0.001
NKPI	Group I/II	18/8	26/12				
	Group III/IV	20/23	29/33	2.162	<0.001	2.036	0.001
GPS	0	32	46				
	1	22	32				
	2	15	22	2.660	<0.001	3.052	<0.001

*Abbreviations*: *PFS* progression-free survival, *OS* overall survival, *HR* hazard ratio, *ECOG* Eastern Cooperative Oncology Group, *LD* lactate dehydrogenase, *LN* lymph node, *EBV* Epstein-Barr virus, *IPI* International Prognostic Index, *NKPI* NK/T-cell Lymphoma Prognostic Index, *CRP* C-reactive protein, *GPS* Glasgow Prognostic Score.

### Serum CXCL13 and the GPS

**Figure 2:**
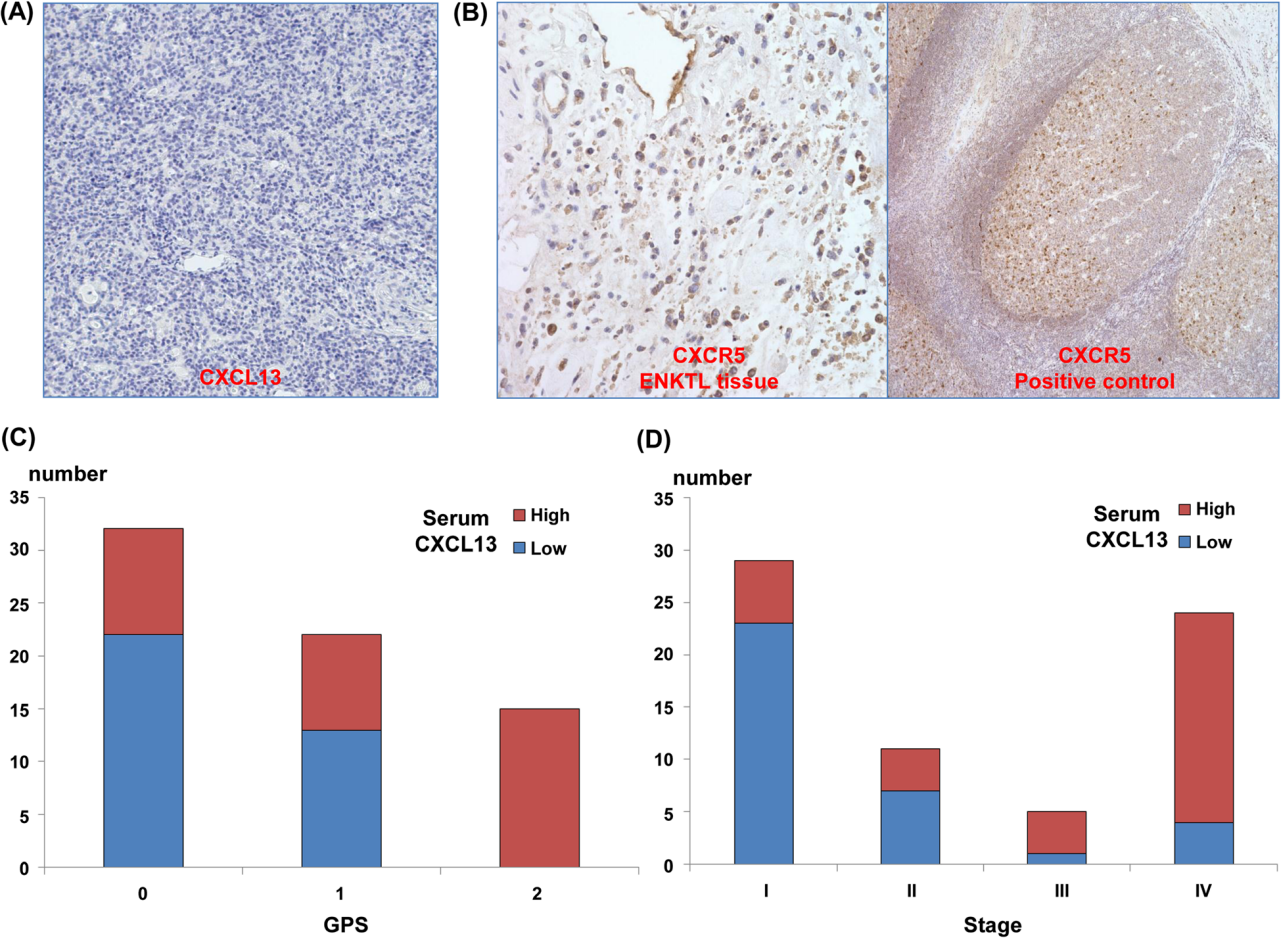
Tissue expression of CXCL13 and CXCR5, and its correlation with GPS and stage. **(A)** Representative ENKTL case with negative CXCL13 staining (×400). **(B)** CXCR5 antibody stains the cytoplasm of ENKTL tumor cells and B-cells in a normal germinal center. **(C)** There were significantly more patients with high CXCL13 in the GPS 2 group than in the GPS 0 or 1 groups (*P* < 0.05). **(D)** More patients have high CXCL13 with stage III/IV disease than do patients with stage I/II (*P* < 0.05).

The mean value of CXCL13 was 155.2 pg/mL (standard deviation (SD): 406.7 pg/mL). There was only one patient with an undetectable CXCL13 level. The median value was 29.1 pg/mL (range: 0.0–2517.6 pg/mL). Patients were categorized into high (CXCL13 level > median value, *n* = 34) and low (CXCL13 level ≤ median value, *n* = 35) CXCL13 groups. The high CXCL13 group had a higher frequency of PFS events (27/34, 79%), including relapse/progression or death, than did the low CXCL13 group (13/35, 37%). Therefore, high levels of CXCL13 were significantly associated with worse OS and PFS (Figure 1C, D). Immunohistochemical staining for CXCL13 from 30 patients revealed that all of the tumor cells were negative for CXCL13 (Figure 2A). The expression of CXCL13 was also negative in stromal cells of the tumor tissue although normal lymphocytes adjacent tumor tissue showed positive expression of CXCL13. In contrast, the tumor cells did express CXCR5 (Figure 2B). The serum CXCL13 was significantly associated with risk stratification by the GPS. In particular, all patients from the GPS 2 group had serum levels of CXCL13 that are higher than the median value (*P* < 0.001, Figure 2C). The mean serum level from the GPS 2 group 2 (505.5 ± 778.3 pg/mL) was significantly higher than that of the GPS 0 (50.2 ± 74.3 pg/mL) and GPS 1 (69.0 5 ± 106.8 pg/mL) groups. High CXCL13 levels were significantly associated with unfavorable prognostic signs including advanced stage (III/IV, Figure 2D), extranasal presentation, bone marrow invasion, and extranodal involvement (Table 2). In addition, patients classified as high-risk by the IPI or NKPI had significantly higher serum levels of CXCL13 than did those at lower risk (*P* < 0.001). EBV positivity was also significantly associated with CXCL13 (*P* < 0.001). Like CXCL13, the GPS also showed a significant association with unfavorable parameters and high-risk groups of IPI and NKPI (Table 2). However, serum levels of CXCL13 and GPS failed to show independent prognostic values for OS and PFS in the multivariate analysis with clinical parameters (*P* < 0.05, data not shown).

**Table 2:** **The association of CXCL13 and CCL3 with unfavorable parameters**

		**CXCL13**			**GPS**		
**Characteristics**		**Low**	**High**	***P***	**0/1**	**2**	***P***
Age (years)	≤60	32	24		48	8	
	>60	3	10	0.034	6	7	0.005
Ann Arbor stage	I/II	30	10		37	3	
	III/IV	5	24	<0.001	17	12	0.001
Serum LDH	Normal	22	8		28	2	
	Increased	13	26	0.001	26	13	0.009
B symptoms	Absence	25	14		35	4	
	Presence	10	20	0.015	19	11	0.017
LN involvement	Absence	25	15		36	4	
	Presence	10	19	0.029	18	11	0.008
Primary site	Nasal	29	12		38	3	
	Extranasal	6	22	<0.001	16	12	0.001
Bone marrow invasion	No	33	23		49	7	
	Yes	2	11	0.006	5	8	0.001
Extranodal involvement	0/1	29	14		38	5	
	≥2	6	20	<0.001	16	10	0.015
EBV DNA	Non-detectable	29	13		38	4	
	Detectable	6	21	<0.001	16	11	0.006
IPI	L/LI	30	14		42	2	
	HI/H	5	20	<0.001	12	13	<0.001
NKPI	Group I/II	22	4		24	2	
	Group III/IV	13	30	<0.001	30	13	0.036

*Abbreviations*: *LDH* lactate dehydrogenase, *LN* lymph node, *EBV* Epstein-Barr virus, *IPI* International Prognostic Index, *NKPI* NK/T-cell Lymphoma Prognostic Index, *GPS* Glasgow Prognostic Score.

### Subgroup analysis

**Figure 3:**
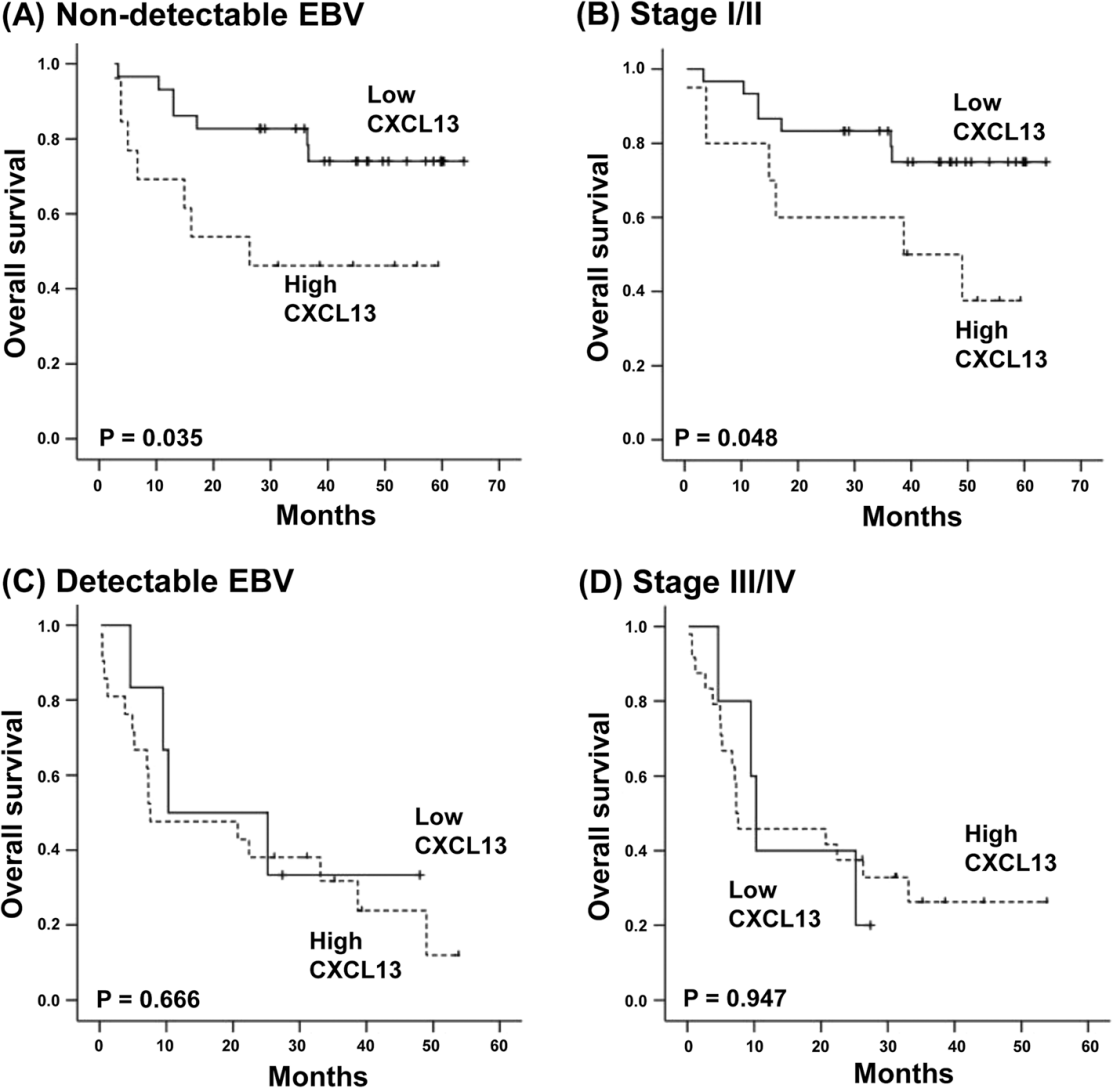
The subgroup analysis for the association of serum CXCL13 with overall survival based on stage and EBV DNA positivity in blood. **(A, B)** High CXCL13 levels are associated with lower overall survival among patients with non-detectable EBV titers and stage I/II disease. **(C, D)** CXCL13 fails to predict prognosis in patients with advanced disease and detectable EBV titers.

**Figure 4:**
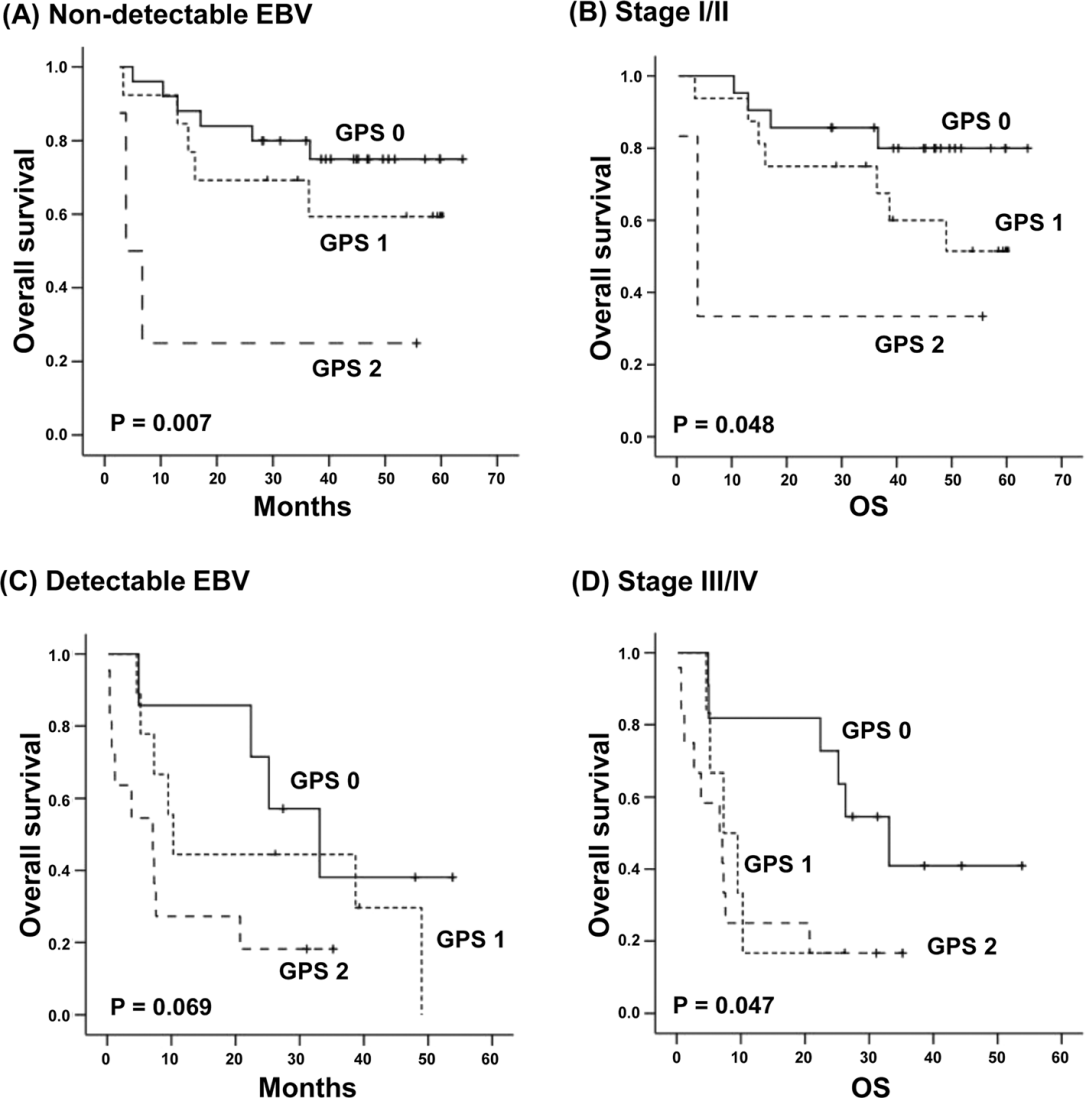
The subgroup analysis for the association of GPS with overall survival based on stage and EBV DNA positivity in blood. **(A, B)** GPS is associated with lower overall survival among patients with non-detectable EBV titers and stage I/II disease. **(C, D)** GPS shows a marginal significance in patients with advanced disease and detectable EBV titers.

As grouping by the serum CXCL13 level and GPS was not independently prognostic for survival outcomes, the subgroup analysis was done according to tumor burden. Patients were dichotomized into high and low tumor burden based on stage and EBV DNA in blood. Elevated CXCL13 levels were associated with poor OS in patients with low tumor burden: those with non-detectable EBV or stages I/II (Figure 3A, B). In contrast, in patients with high tumor burden such as stage III/IV or detectable EBV, the serum CXCL13 was unable to predict prognosis (Figure 3C, D). Grouping by the GPS also showed a significant association with OS in patients with low tumor burden similar to serum CXCL13 level (Figure 4A, B). The association of the GPS with poor OS was less significant in patients with stage III/IV and detectable EBV DNA (Figure 4C, D). The lack of prognostic significance of CXCL13 and GPS in patients with high tumor burden might be related with a strong correlation of elevated CXCL13 and GPS 2 with stage III/IV and detectable EBV in this study (Table 2).

## Discussion

Although a role for inflammation in cancer has long been postulated in the mid-1800s, evidence supporting the association of inflammation with cancer initiation, progression, and mortality has been gathered more recently. The GPS based on CRP and serum albumin reflects the systemic inflammatory response to cancer, and its proven prognostic value in cancer patients augments the contributory role of inflammation to tumor aggressiveness. Our study demonstrated the GPS had a significant association with OS and PFS in ENKTL patients inconsistent with a previous finding [4]. Thus, patients who were given a GPS of 2 showed worse OS and PFS than patients with GPS 0 or 1 (Figure 1A, B). As we hypothesized CXCL13, an inflammatory chemokine might contribute to the prognostic value of GPS, patients with an elevated serum CXCL13 level showed poor survival outcomes (Figure 1C, D). Furthermore, an elevated serum CXCL13 level was strongly correlated with the GPS, especially a score of 2 because all patients with GPS 2 showed an elevated level of CXCL13 (Figure 2C).

CXCL13 is a follicular helper T-cell marker and a known B-cell chemoattractant that plays a role in germinal center formation [25,26]. Prior to this study, there was no data about the prognostic value of serum CXCL13 in ENKTL as well as its relation with the GPS. Our study demonstrated a significant association between serum CXCL13 levels and a high-risk ENKTL classification according to the GPS (*P* < 0.001, Figure 2C). Thus, our findings suggested the increased serum levels of CXCL13 representing inflammatory activity might be associated with unfavorable characteristics of ENKTL such as tumor burden like previous studies reporting the contribution of CXCL13 to solid tumor progression, that is, [6,7]. The immunohistochemistry with tumor tissue showed the positive staining for CXCR5 and negative for CXCL13 on lymphoma cells (Figure 2A, B). This result suggests that the serum CXCL13 might be mostly coming from inflammatory cells, not tumor cells. Although there is no direct data about the interaction of CXCL13 with CXCR5 in lymphoma cells of ENKTL, a previous *in vitro* study with AIDS-related non-Hodgkin lymphoma cell lines demonstrated migration of lymphoma cells expressing CXCR5 toward CXCL13 [27]. Given that AIDS-related NHL is frequently related with EBV infection, this CXCL13/CXCR5 interaction might be present in ENKTL like AIDS-related NHL. Thus, the association of serum CXCL13 with stage of ENKTL might be influenced by the major contributory effect of CXCL13 to cell migration and invasion. However, further studies are required to clarify the underlying mechanism by which CXCL13/CXCR5 interaction is associated with aggressiveness of ENKTL.

The grouping by serum levels of CXCL13 as well as the GPS failed to show an independent prognostic value for survival outcomes in this study unlike a previous study demonstrating the superior independent prognostic value of the GPS to IPI and NKPI in 164 patients with ENKTL [4]. These findings might be related with a relatively smaller number of patients in our study (*n* = 69) than the previous study. However, the strong correlation of serum CXCL13 with the GPS as well as high tumor burden such as stage and EBV DNA might influence their prognostic value in the multivariate analysis. On the other hand, in the subgroup analysis based on tumor burden, grouping by the serum level of CXCL13 and GPS discriminated patients with poor survival outcomes among those with stage I/II disease and/or with non-detectable EBV DNA (Figures 3 and 4). Following this, patients with elevated CXCL13 levels may be at a higher risk of treatment failure like patients with GPS 2. Our study results were from ENKTL patients that were registered in our prospective cohort study, and all patients were treated with non-anthracycline-based chemotherapy and/or concurrent chemoradiotherapy. Considering the majority of prior studies were retrospective in nature and included patients who were treated with anthracycline-based chemotherapy [1,28], our findings may be relevant to patients who have been treated with the current standard of care. The association of serum CXCL13 with GPS and survival outcomes of ENKTL patients might suggest the possibility of CXCL13 as a prognostic indicator and therapeutic target in patients with ENKTL. Therefore, future studies with larger patient populations should be warranted to confirm the prognostic value of serum CXCL13.

## Abbreviations

GPS: Glasgow Prognostic Score
ENKTL: Extranodal NK/T-cell lymphoma
CXCL13: C-X-C motif ligand 13
CXCR5: C-X-C chemokine receptor 5
CRP: C-reactive protein
CCRT: Concurrent chemoradiotherapy
SMILE: Steroid, methotrexate, ifosfamide, L-asparaginase, and etoposide
IPI: International Prognostic Index
NKPI: NK Prognostic Index
PFS: Progression-free survival
OS: Overall survival
