# Erratum to: The serum CXCL13 level is associated with the Glasgow Prognostic Score in extranodal NK/T-cell lymphoma patients

**DOI:** 10.1186/s13045-015-0200-y

**Published:** 2015-10-08

**Authors:** Seok Jin Kim, Kyung Ju Ryu, Mineui Hong, Young Hyeh Ko, Won Seog Kim

**Affiliations:** Division of Hematology and Oncology, Department of Medicine, Samsung Medical Center, Sungkyunkwan University School of Medicine, Seoul, Korea; Samsung Biomedical Research Institute, Samsung Medical Center, Seoul, Korea; Department of Pathology, Kangnam Sacred Heart Hospital, Hallym University, Seoul, Korea; Department of Pathology, Samsung Medical Center, Sungkyunkwan University School of Medicine, Seoul, Korea; Department of Health Sciences and Technology, SAIHST, Sungkyunkwan University School of Medicine, Seoul, Korea

Unfortunately, the original version of this article [1] contained an error. The legend for Table 2 was included incorrectly. The corrected legend is ‘The association of CXCL13 with unfavourable parameters’.
